# Systematic Review and Meta-Analysis of Total Elbow Arthroplasty in Outpatient Versus Inpatient Settings

**DOI:** 10.7759/cureus.72378

**Published:** 2024-10-25

**Authors:** Blake Martin, Yolanda Gutierrez, Joshua Nwose, Juan C Lopez-Alvarenga, Robert Ablove

**Affiliations:** 1 Medicine, University of Texas Rio Grande Valley, Edinburg, USA; 2 Orthopedics, University of Texas Rio Grande Valley, Edinburg, USA; 3 Population Health and Biostatistics, University of Texas Rio Grande Valley, Edinburg, USA; 4 Orthopedic Surgery, Jacobs School of Medicine and Biomedical Sciences, Buffalo, USA

**Keywords:** arthroplasty, elbow, inpatient, joint replacement, meta-analysis, outpatient

## Abstract

Total joint arthroplasties, including total elbow arthroplasty (TEA), are growing in number and shifting toward a younger age group. These procedures are also moving more toward the outpatient setting compared to previous years. We are conducting this study to update and summarize the current knowledge about the outcomes of TEA in outpatient versus inpatient settings. We conducted a systematic review and meta-analysis using the Preferred Reporting Items for Systematic Reviews and Meta-Analyses (PRISMA) framework to compare TEA outcomes in outpatient versus inpatient settings. Electronic database searches were performed using PubMed, Cochrane Database of Systematic Reviews, Cochrane Central Register of Controlled Trials, and Google Scholar. Previous studies deemed eligible for this study focused on inpatient cohorts and outpatient cohorts or compared inpatient and outpatient cohorts receiving TEA. All data used was obtained from the studies that were included. Three studies were deemed eligible and included a total of 1,634 patients, with 1,048 being inpatient and 586 being outpatient. There was a significant increase in total (any) complication rate (log of odds ratio (lnOR): 1.02, 95% CI: 0.35 to 1.68), adverse discharge (lnOR: 1.07, 95% CI: 0.22 to 1.92), and surgical site infection (lnOR: 0.88, 95% CI: 0.05 to 1.71) in the inpatient setting compared to the outpatient setting. There was no significant difference between outpatient and inpatient settings in regard to readmissions (lnOR: 0.85, 95% CI: -0.95 to 2.66), urinary tract infections (UTI)/renal complications (lnOR: 0.04, 95% CI: -0.09 to 0.17), pneumonia/respiratory failure (lnOR: 0.61, 95% CI: -0.29 to 1.51), deep vein thrombosis (DVT)/pulmonary embolism (lnOR: 0.07, 95% CI: -1.31 to 1.44), sepsis (lnOR: 0.86, 95% CI: -0.50 to 2.21), and wound dehiscence (lnOR: 0.55, 95% CI: -0.58 to 1.68). Our results reveal that with careful patient selection, current surgical techniques, and pain control methods, TEA may be performed in the outpatient setting with less risk of complications and lower financial burden compared to inpatient TEA.

## Introduction and background

Total elbow arthroplasty (TEA) is increasing in prevalence [1]. Pasternack et al. state that the number of primary TEAs performed has increased by 248% from 1993 to 2007 [1]. They also state that from 2007 to 2011, the number of TEAs performed in the United States increased steadily, at a rate of about 600-700 additional procedures per year [1]. However, although knee and hip arthroplasty have mostly transferred to the outpatient setting, TEA remains a primarily inpatient procedure [2]. TEA is a common surgical procedure used in the management of various orthopedic conditions with the majority being arthritis related such as advanced rheumatoid arthritis, posttraumatic arthritis, osteoarthritis, as well as unfixable fracture in older patients [3]. Total elbow prostheses have improved through the years with linked, unlinked, and convertible types now available for use [3]. However, there are various long-term complications that remain a problem such as infection, instability, aseptic loosening, and periprosthetic fracture [3]. In comparison to total knee arthroplasty and total hip arthroplasty, TEA is relatively rare [3]. Therefore, to avoid iatrogenic errors, surgeons should carefully review the previous literature on TEA [3].

The term outpatient typically refers to patients who go home the same day or within 24 hours after the surgery. The term inpatient typically refers to patients who stay one or more nights at the hospital after a procedure is performed. The terms inpatient and outpatient vary based on the hospital or surgical center where the procedure occurs. One reason for a push to move toward the outpatient setting is the potential for profound financial savings. According to Pasternack et al., outpatient arthroplasty is accompanied by substantial monetary savings for the healthcare system [1]. A study by Baxter et al. identified 307 outpatient and 414 inpatient TEA procedures over a nine-year period, and in terms of cost, the median inpatient TEA was more expensive than outpatient TEA ($26,817 vs $18,412) [2].

Total joint arthroplasties, including TEA, are growing in number and shifting toward a younger age group [1,4]. These surgical procedures are also moving more toward the outpatient setting compared to previous years [1]. The ever-advancing field of medicine with alterations in surgical procedures, preoperative preparation, postoperative care, and changes in the number of outpatient surgeries may have strengthened or may show controversy regarding previous studies on this topic. To our knowledge, we are conducting the first systematic review and meta-analysis on TEA in the outpatient vs. inpatient setting. We are conducting this study to update and summarize the current knowledge about the outcomes of TEA in outpatient versus inpatient settings to allow for the best possible care when performing this procedure. We hypothesized that outpatient TEA would have less complication rates compared to inpatient TEA.

## Review

Methods and materials

Data Collection

We conducted a systematic review and meta-analysis using the Preferred Reporting Items for Systematic Reviews and Meta-Analyses (PRISMA) framework to compare TEA outcomes and complications in outpatient versus inpatient settings [5]. This study was IRB exempt as the studies (data) used were publicly available. Electronic database searches were performed using PubMed (49), Cochrane Database of Systematic Reviews (1), Cochrane Central Register of Controlled Trials (14), and Google Scholar (10,700) from their dates of establishment to June 2024. Our search was conducted by combining the terms “outpatient OR ambulatory surgery OR day-case AND inpatient OR overnight stay” AND “elbow arthroplasty OR elbow replacement” when searching in the MeSH, title, abstract, and keywords fields.

Study Criteria

Studies deemed eligible for this systematic review and meta-analysis focused on inpatient cohorts, outpatient cohorts, or compared inpatient and outpatient cohorts receiving TEA. The inpatient/outpatient variable in our study relies on individual hospital and surgical center definitions, although outpatient procedures are generally performed at an ambulatory surgery center instead of a hospital. The total joint arthroplasty cohort in this study consisted only of TEA. Unicompartmental elbow replacement or elbow resurfacing patients were excluded. Studies must have had postoperative complications reported. The study with the longest follow-up period was included if multiple studies focused on the same cohort. Only publications written in English that included human subjects were included. Expert opinions, reviews, case reports, forum presentations, and editorials were excluded. We used the Newcastle Ottawa Scale to assess the quality of studies used, and potential sources of bias were accounted for when selecting studies and interpreting the data [6].

Study Design

All data used was obtained from the studies’ that were included. The characteristics analyzed for each study included study year, number of patients undergoing TEA, and number of inpatients or outpatients. Corresponding authors were contacted to obtain data not included in their studies. The preoperative demographics analyzed were age and sex. Preoperative demographics were analyzed using two studies. The preoperative comorbidities were diabetes, pneumonia/chronic obstructive pulmonary disease (COPD), and smoking. Diabetes was analyzed using all three studies, whereas the other preoperative comorbidities were analyzed using only two studies. The outcomes reported were any complication, readmissions, reoperations, urinary tract infection (UTI)/renal complications, pneumonia/respiratory failure, deep vein thrombosis (DVT)/embolism, stroke, myocardial infarction (MI), cardiac arrest, sepsis, wound infection, and wound dehiscence. The outcome “any complication” was analyzed by utilizing all three studies. However, the data for the specific complications was not available for all three studies, even after reaching out to the corresponding authors. Therefore, specific complications were analyzed by utilizing only two of the eligible studies.

Data Analyses

We obtained the odds ratio (OR) (95% CI) from the studies. The meta-analysis was performed with a random effects model, and I2 was calculated to analyze the heterogeneity. A funnel plot with 95% CI was calculated to analyze the possibility of publication bias. However, the results should be considered with caution due to the small sample size. All analyses were performed with StataNow 18.5 (StataCorp College Station, TX).

Results

Quality of Studies

**Figure 1:**
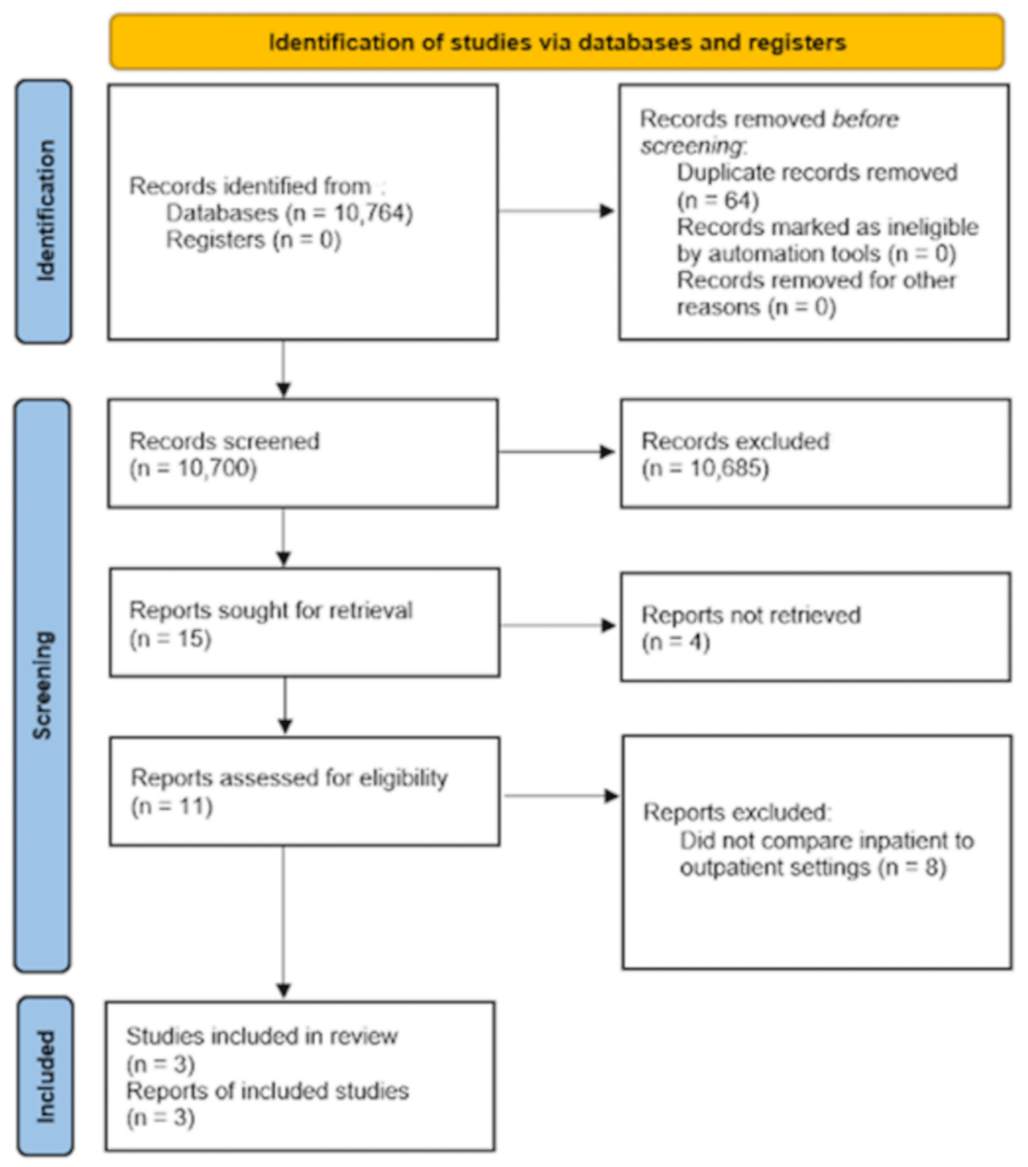
2020 PRISMA flow chart for systematic review and meta-analysis of TEA From Page et al. [5]. PRISMA: Preferred Reporting Items for Systematic Reviews and Meta-Analyses, TEA: total elbow arthroplasty.

**Figure 2:**
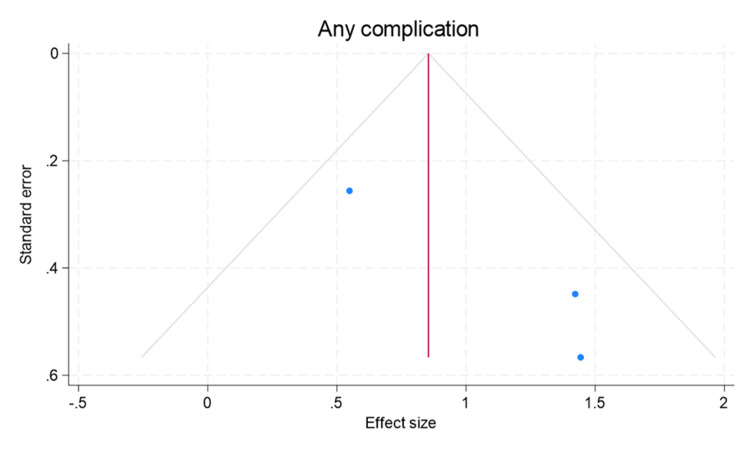
Funnel plot for analyzation of publication bias using the "any complication" rate

A total of 10,764 studies were identified by searching four electronic databases using the 2020 PRISMA flow chart (Figure 1) [5]. Of this starting number, 64 duplicates were found and removed. A total of 10,685 records were excluded from the 10,700 that were screened, leaving 15 reports to be sought for retrieval. Of the 15 reports, four were not able to be retrieved. The remaining 11 reports were analyzed for eligibility, and three of them were deemed eligible for the systematic review and meta-analysis [2,7,8]. The studies included a total of 1,634 patients, with 1,048 being inpatient and 586 being outpatient. The demographics and characteristics of the studies were analyzed (Table 1). All three studies were performed retrospectively as retrospective cohorts. The Newcastle Ottawa Scale was used to determine the quality of the included studies with a score of 7 or greater indicating a high-quality study, whereas scores less than 7 indicating a low-quality study (Table 2) [6]. There was no significant publication bias in the total complication rates for TEA when a tunnel plot was performed (Figure 2).

**Table 1:** Characteristics and demographics of included studies RC: retrospective cohort.

Author	Year	Study Period	Country	Study Type	Total Patients	Number of Patients		Follow-Up Period
						Outpatient	Inpatient	
Baxter et al. [2]	2023	2009-2017	USA	RC	721	307	414	90 days
Furman et al. [7]	2020	2007-2017	USA	RC	445	114	331	30 days
Momtaz et al. [8]	2023	2016-2020	USA	RC	468	165	303	30 days

**Table 2:** Newcastle Ottawa Quality Assessment Scale

Item	Baxter et al. [2]	Momtaz et al. [8]	Furman et al. [7]
Selection	4	4	4
1. Representativeness of the intervention cohort			
2. Selection of the non-intervention cohort			
3. Ascertainment of intervention			
4. Demonstration that outcome of interest was not present at start of study			
Comparability	2	0	1
Comparability of cohorts on the basis of the design or analysis			
Outcome	3	3	3
1. Assessment of outcome			
2. Was follow-up long enough for outcome to occur			
3. Adequacy of follow-up of cohorts			
Total	9	7	8

Study Characteristics

The mean patient age in the inpatient setting for TEA ranged from 64.1 (±14.1) to 65.1 (±13.5) years old, whereas the mean age in the outpatient setting ranged from 63.7 (±14.2) to 65.3 (±12.2) years old. The mean age was gathered from the studies by Baxter et al. and Furman et al., and the difference in mean age was not significant [2,7]. In the inpatient group, 56.1% of the population were men, whereas in the outpatient group, 60.1% of the population were men. The sex differences in the outpatient versus the inpatient setting were determined by using the studies by Baxter et al. and Furman et al., and there were no significant differences in sex between outpatient and inpatient populations [2,7]. The follow-up period for the included studies ranged from 30 to 90 days [2,7,8]. The outpatient definition in our study relies on individual hospital and surgical center definitions.

**Figure 3:**
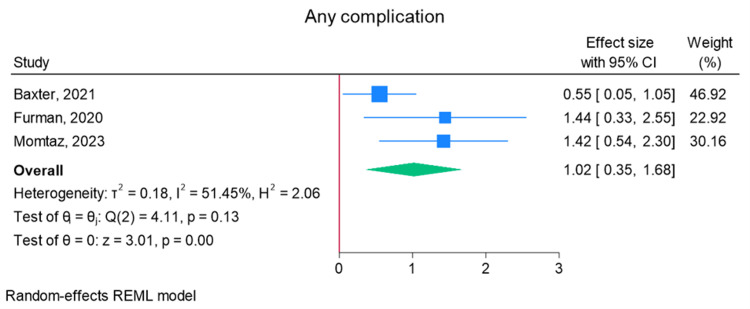
Random effects model for any complication Baxter et al. [2], Furman et al. [7], and Momtaz et al. [8]. REML: restricted maximum likelihood.

**Figure 4:**
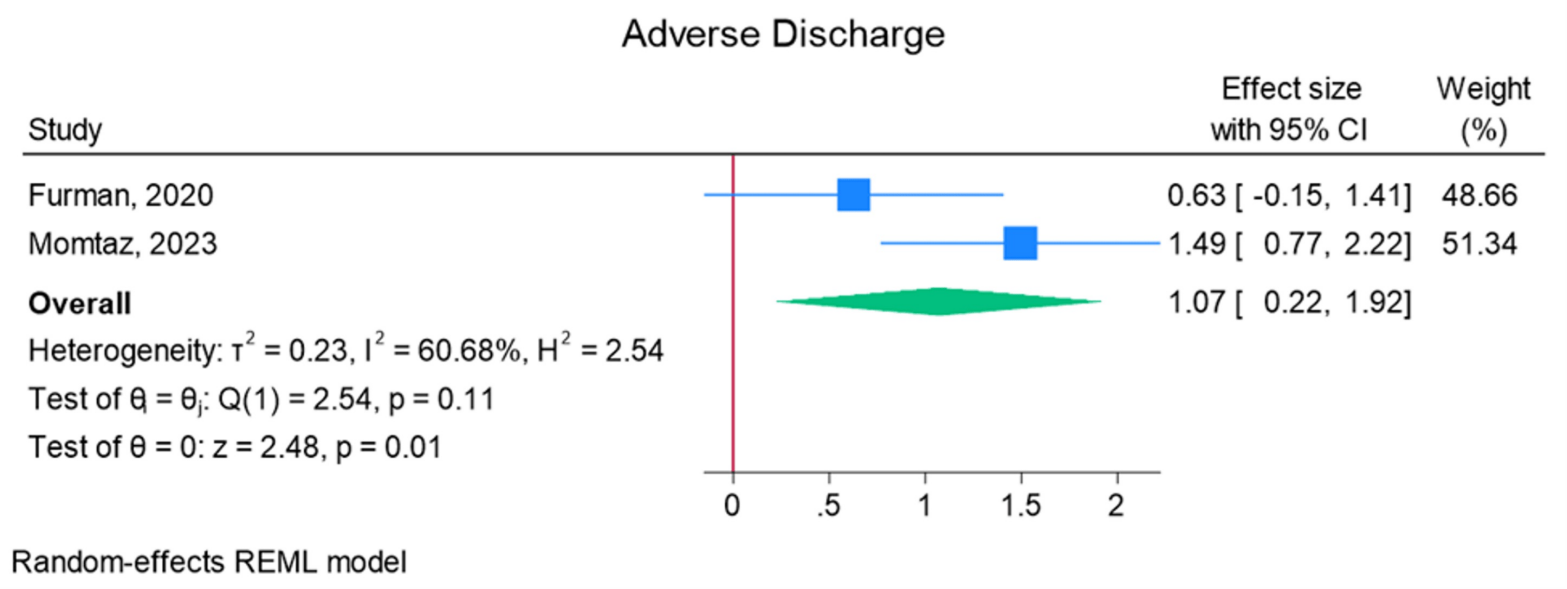
Random effects model for adverse discharge Furman et al. [7] and Momtaz et al. [8]. REML: restricted maximum likelihood.

**Figure 5:**
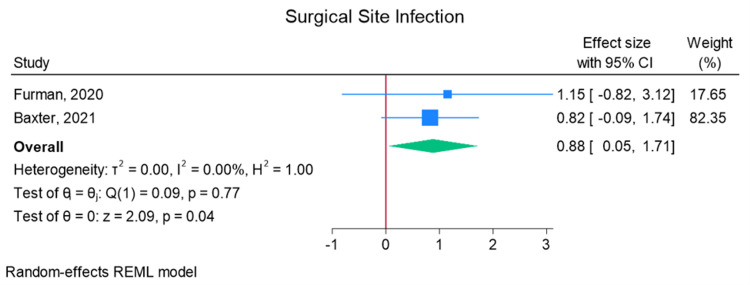
Random effects model for surgical site infection Baxter et al. [2] and Furman et al. [7]. REML: restricted maximum likelihood.

**Figure 6:**
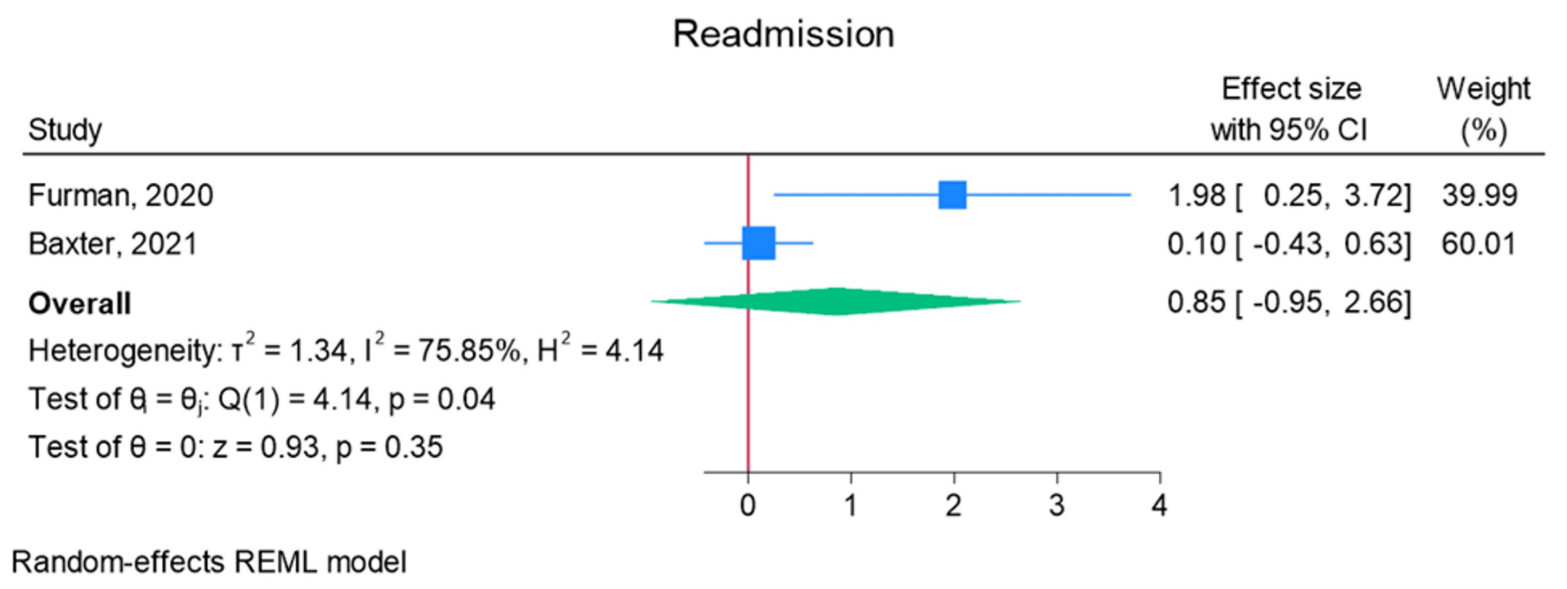
Random effects model for readmission Baxter et al. [2] and Furman et al. [7]. REML: restricted maximum likelihood.

**Figure 7:**
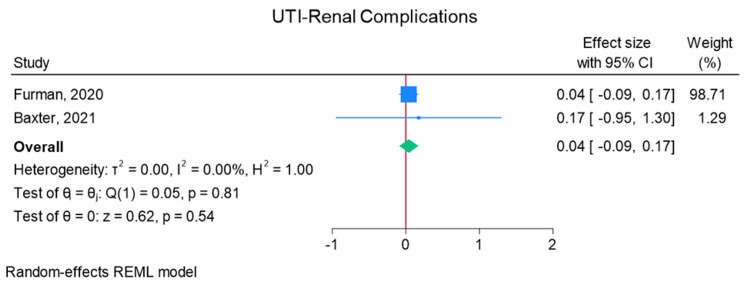
Random effects model for UTI/renal complications Baxter et al. [2] and Furman et al. [7]. UTI: urinary tract infections, REML: restricted maximum likelihood.

**Figure 8:**
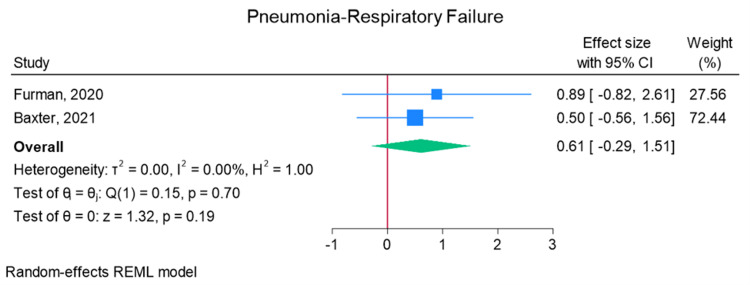
Random effects model for pneumonia/respiratory failure Baxter et al. [2] and Furman et al. [7]. REML: restricted maximum likelihood.

**Figure 9:**
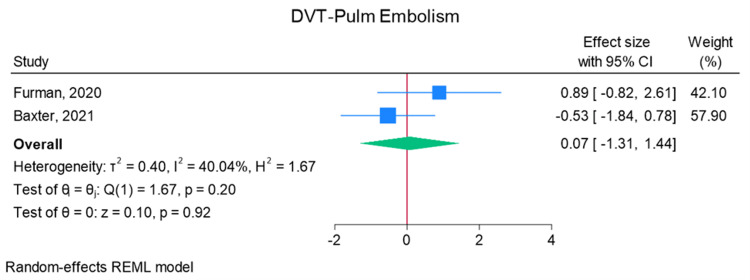
Random effects model for DVT/pulmonary embolism Baxter et al. [2] and Furman et al. [7]. DVT: deep vein thrombosis, REML: restricted maximum likelihood.

**Figure 10:**
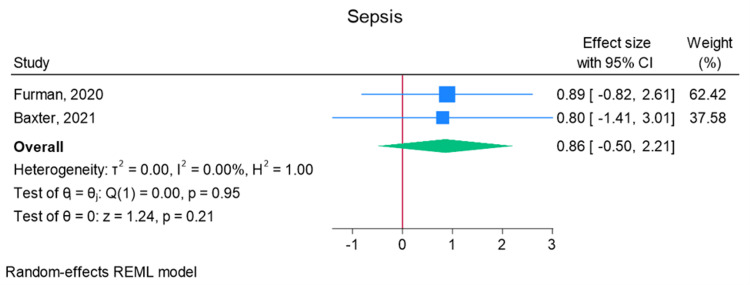
Random effects model for sepsis Baxter et al. [2] and Furman et al. [7]. REML: restricted maximum likelihood.

**Figure 11:**
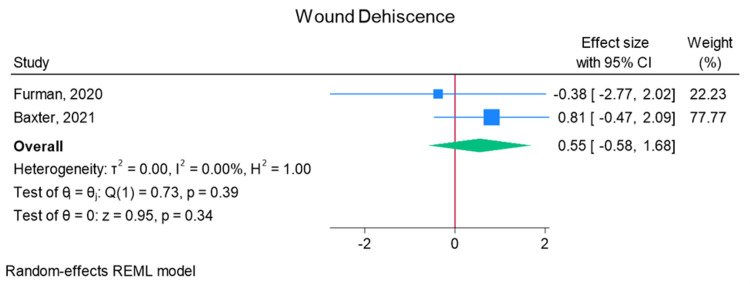
Random effects model for wound dehiscence Baxter et al. [2] and Furman et al. [7]. REML: restricted maximum likelihood.

Total elbow arthroplasty outcomes: In the random effects models, greater than 0 favors outpatient, whereas less than 0 favors inpatient. Adverse discharge is defined as any non-home discharge. There was a significant increase in total (any) complication rate for inpatients compared to outpatients (log of odds ratio (lnOR): 1.02, 95% CI: 0.35 to 1.68, heterogeneity (I2) = 51.45%, P = 0.00) (Figure 3). There was also a significant increase in adverse discharge (lnOR: 1.07, 95% CI: 0.22 to 1.92, I2 = 60.68%, P = 0.01) and surgical site infection (lnOR: 0.88, 95% CI: 0.05 to 1.71, I2 = 0.00%, P = 0.04) in the inpatient setting compared to the outpatient setting (Figures 4, 5). There was no significant difference between outpatient and inpatient settings in regard to readmissions (lnOR: 0.85, 95% CI: -0.95 to 2.66, I2 = 75.85%, P = 0.35), UTI/renal complications (lnOR: 0.04, 95% CI: -0.09 to 0.17, I2 = 0.00%, P = 0.54), pneumonia/respiratory failure (lnOR: 0.61, 95% CI: -0.29 to 1.51, I2 = 0.00%, P = 0.19), DVT/pulmonary embolism (lnOR: 0.07, 95% CI: -1.31 to 1.44, I2 = 40.04%, P = 0.92), sepsis (lnOR: 0.86, 95% CI: -0.50 to 2.21, I2 = 0.00%, P = 0.21), and wound dehiscence (lnOR: 0.55, 95% CI: -0.58 to 1.68, I2 = 0.00%, P = 0.34) (Figures 6-11).

Discussion

While more complicated cases of TEA may be performed in an inpatient setting, outpatient TEA is becoming more common. Although outpatient TEA has reduced costs, there is minimal evidence regarding the safety of this procedure in outpatient compared to inpatient settings. In this study, we sought to perform a systematic review and meta-analysis on eligible studies to determine the difference, if any, in outcomes regarding TEA in the outpatient versus the inpatient setting. We hypothesized that outpatient TEA would have better outcomes with lower complication rates compared to inpatient TEA.

Our results show that there was a significant increase in total (any) complication rate, adverse discharge, and surgical site infection for inpatients compared to outpatients. However, there was no significant difference between outpatient and inpatient settings in regard to readmissions, UTI/renal complications, pneumonia/respiratory failure, DVT/pulmonary embolism, sepsis, and wound dehiscence. These results show that not only is outpatient TEA just as safe as inpatient TEA but outpatient TEA is also shown to be safer in regard to complication rate, adverse discharge, and surgical site infections so long as efficient patient selection is in place. In addition to being a safer environment for TEA, the outpatient setting also offers financial benefits. Baxter et al. state that the median inpatient TEA is significantly more expensive than outpatient TEA ($26,817 vs $18,412) [2]. However, Baxter et al. state that the median cost for occupational therapy within 90 days of surgery is slightly higher for outpatient TEA patients ($687 vs $571) [2]. This combination of increased safety and financial efficiency makes outpatient TEA a favorable choice for individuals deemed eligible for the outpatient setting.

For satisfactory total joint arthroplasty outcomes, it is essential that effective preoperative patient selection is performed to decide if an outpatient protocol is suitable [9]. In our study, there was a slightly higher percentage of men in the outpatient group compared to the inpatient group and the mean age was similar between outpatient and inpatient groups. Compared to men undergoing TEA, women had significantly lower mortality, 0.1% vs. 0.4% (p=0.03); lower proportion were discharged to home, 81.9% vs. 89.6% (p<0.0001); and fewer had index hospital stay above the median, 30.0% vs. 33.0% (p=0.01) [10]. So, although women tend to have better outcomes following TEA, there was a lower percentage of women that underwent outpatient TEA in our study, suggesting that sex is not a major factor compared to other patient characteristics and comorbidities when the decision of outpatient or inpatient TEA is being made. Although the mean age in our study was similar between outpatient and inpatient settings, younger adults are generally healthier than older adults, suggesting that the age of an adult is not a major factor when deciding whether to perform TEA in the outpatient or inpatient setting. Based on previous studies, some common inclusion criteria for performing outpatient total joint replacement include living within one hour from the hospital and body mass index (BMI) less than 40 kg/m [9]. An abundance of comorbidities including smoking, cardiovascular disease, diabetes, and corticosteroid use may make patients ineligible for total joint arthroplasty or outpatient total joint arthroplasty as there is an increased risk of postoperative complications [9]. Despite this, it has been shown in a study by Xu et al. that patients who have significant medical comorbidities may still be safely discharged as outpatients; however, you must consider other factors including support at home after surgery [9]. Xu et al. also showed that anesthetic related side effects such as nausea and hypotension are the most common reason for delayed discharge; therefore, shorter acting agents and aggressive management of these symptoms may be beneficial by reducing length of stay [9]. Adequate pain control and early mobilization are also essential when planning a same-day discharge according to Xu et al. [9].

An explanation for the increased risk of complications, adverse discharge, and surgical site infection in the inpatient setting as shown in this study is that patients who have higher risk such as those with certain comorbidities, and adolescents and children are typically chosen to undergo the procedure in the hospital (inpatient) setting. This may be done to maximize the safety of the patient because the hospital generally has more medical equipment, supplies, and staff than surgery centers. With the risk of complications being higher in certain individuals, it may be beneficial to have access to everything you may need if a complication arises. Thus, with more at-risk individuals being assigned to inpatient TEA, there is more likely to be an increased number of complications when compared to generally more healthy outpatient individuals.

There are some limitations to our study. First, our study utilized a small number of studies that provided a relatively small sample size. Future meta-analysis studies should look to use a larger number of studies to increase the power of the study results. Secondly, a funnel plot could not be created to search for publication bias in all outcomes that we measured, due to the lack of available data in the studies used. Thirdly, the previous studies included in our meta-analysis used a variety of surgical techniques, preoperative and postoperative care, and patient selection criteria which could increase heterogeneity. Lastly, the definition of an outpatient differed between analyzed studies, from same-day discharge to discharge within 24 hours. Thus, outpatient individuals in some studies may not be defined as outpatients in other studies. Future studies on TEA in inpatient versus outpatient settings should consider using similar surgical techniques, pre- and postoperative care, patient selection criteria, and similar definitions of "outpatients" to allow for better comparison of studies.

## Conclusions

This meta-analysis showed that there was a significant increase in total (any) complication rate, adverse discharge, and surgical site infection for inpatients compared to outpatients. However, there was no significant difference between outpatient and inpatient settings in regard to readmissions, UTI/renal complications, pneumonia/respiratory failure, DVT/pulmonary embolism, sepsis, and wound dehiscence. These results reveal that with careful patient selection, current surgical techniques, and pain control methods, TEA may be performed in the outpatient setting with less risk of complications and lower financial burden compared to inpatient TEA. Further studies should be performed to strengthen or dispute current literature and increase the power of future systematic reviews and meta-analysis studies.
